# Diagnosis and Guided Surgical Management of a Mandibular Buccal Bifurcation Cyst: A Case Report

**DOI:** 10.1155/crid/5146322

**Published:** 2026-08-18

**Authors:** Adrian Reyes, Ishaan Goel, Steven Kaltman, Yehuda Benjamin, Catalina Mendez de la Espriella, Rodrigo R. Amaral

**Affiliations:** ^1^ Department of Endodontics, Nova Southeastern University, Fort Lauderdale, Florida, USA, nova.edu; ^2^ Department of Oral and Maxillofacial Surgery, Nova Southeastern University, Fort Lauderdale, Florida, USA, nova.edu

**Keywords:** differential diagnoses, mandibular buccal bifurcation cyst, periapical diseases, surgical guide

## Abstract

Mandibular buccal bifurcation cyst (MBBC) is a rare inflammatory odontogenic cyst that frequently mimics chronic periapical pathosis, leading to potential misdiagnosis and unnecessary endodontic treatment. This case report describes the diagnosis and management of an MBBC associated with a vital mandibular left second molar (#18) in a healthy 16‐year‐old female, highlighting the value of pulp vitality testing, cone beam computed tomography (CBCT), and a custom 3D‐printed surgical guide for conservative management. The patient presented with buccal bony expansion and a 12‐mm periodontal probing depth with purulent exudate. The tooth responded normally to sensibility testing, with no history of caries or trauma. Interpretation of CBCT images revealed a well‐corticated mid‐root radiolucency with an expansile pattern and a well‐corticated buccal lesion originating from the buccal cortex with an intact lamina dura. A custom surgical guide fabricated from intraoral scans and CBCT data enabled precise cyst enucleation while preserving tooth vitality. Histopathology showed fibrovascular tissue with a dense chronic inflammatory infiltrate composed of lymphocytes, plasma cells, macrophages, and multinucleated giant cells. The diagnosis of an MBBC was based on clinical, radiographic, and histological findings, enabling preservation of the tooth with a minimally invasive approach. At the 1‐year follow‐up, the tooth remained vital, with normal probing depths and satisfactory bone healing on CBCT compared with the initial presentation. This case report emphasizes the need to consider MBBC in the differential diagnosis of radiolucent periapical lesions. Early detection, along with evidence‐based management and guided surgery, is essential to avoid misdiagnosis and unnecessary endodontic treatment.

## 1. Introduction

The majority of periapical radiolucencies in the jaws are attributable to inflammatory processes that occur secondary to pulpal necrosis, which arises from either bacterial infection or trauma [1, 2]. Approximately 90% of radiolucent or hypodense jawbone lesions provisionally diagnosed radiographically as chronic endodontic pathologies are either periapical cysts or periapical granulomas [3]. This high prevalence underscores the diagnostic value of correlating radiographic findings with pulp sensibility testing and clinical assessment.

Several studies have shown that both benign and malignant lesions can clinically and radiographically mimic periapical endodontic lesions, such as periapical cysts and granulomas. Notably, histopathological analysis reveals that approximately 4% of cases initially diagnosed as chronic periapical disease are unrelated to pulpal pathology, highlighting the importance of an accurate, definitive diagnosis [4].

Mandibular buccal bifurcation cyst (MBBC) exhibits characteristic clinical and radiographic features, including association with a vital mandibular first or second molar in pediatric patients, localized swelling in the affected region, and buccal tilting of the involved molars with root apices oriented toward the lingual cortical plate. Radiographically, the lesion presents as a buccal radiolucency partially or completely encompassing the roots, whereas the periodontal ligament space and lamina dura remain intact. A periosteal reaction on the buccal surface, ranging from a single layer to an onion skin appearance, is frequently observed [5].

Management of MBBC remains a subject of debate, with proposed approaches including the extraction of the tooth involved, followed by curettage of the lesion [6–8] or enucleation and curettage while preserving the affected tooth [9–11]. For endodontists, recognizing MBBC is particularly important because its clinical and radiographic presentations often mimic chronic periapical disease.

This case report is aimed at enhancing clinicians′ recognition of MBBC by outlining its key clinical and radiographic features and presenting an evidence‐based management and guided‐surgical approach that prioritizes surgical planning, accuracy, tooth preservation, and optimal patient care.

## 2. Case Report

This case report has been presented in accordance with the CARE guidelines for case reports [12].

The present case describes a healthy 16‐year‐old female who was referred to the Oral and Maxillofacial Surgery (OMFS) Department at Nova Southeastern University by an outside private practice dentist for the extraction of Tooth #18 due to a concerning radiographic lesion. There was no prior history of dental trauma in the area and no symptoms reported at the time of initial evaluation. After surgical evaluation, she was referred to the Endodontic Department for re‐evaluation.

**Figure 1:**
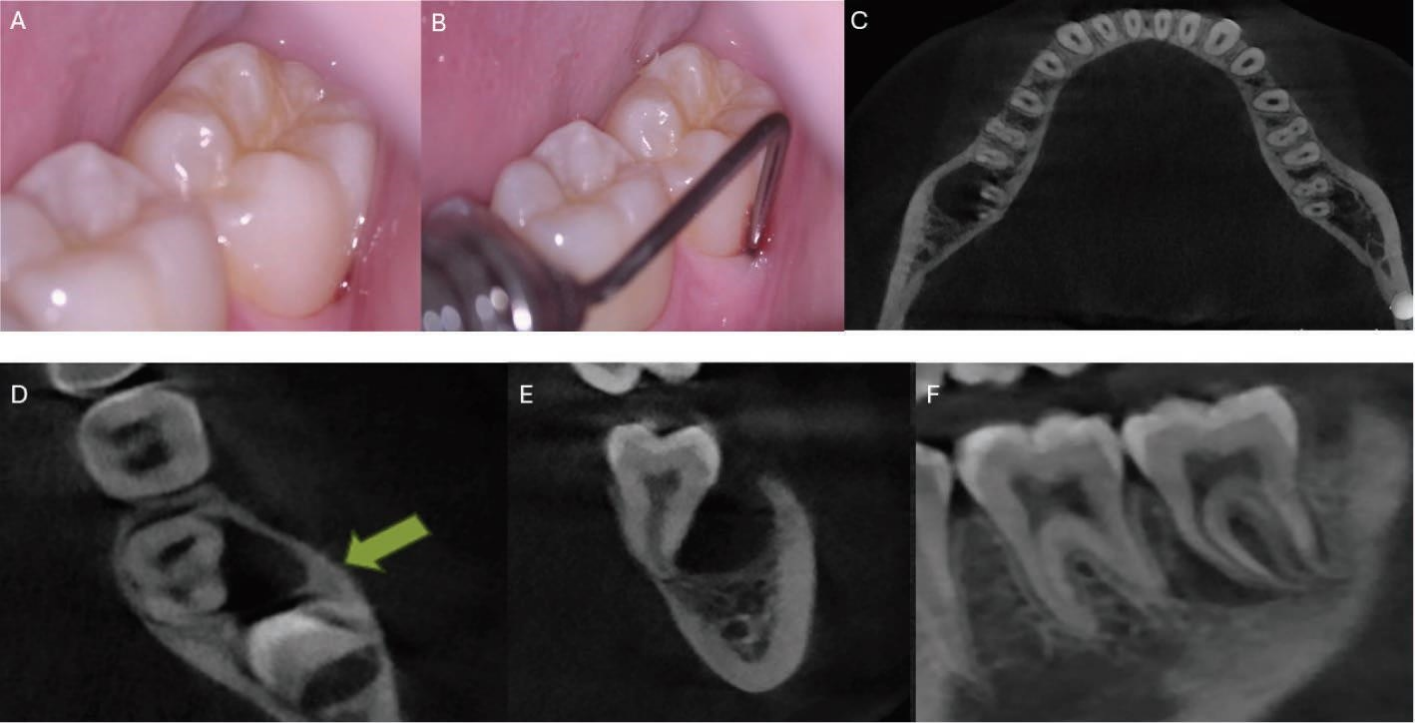
Preoperative CBCT images and clinical photos of Teeth #18 and #19. (A) Photographs of Teeth #18 and #19 at clinical evaluation. (B) The second molar, buccal tilting of the crown, virgin tooth with apparently healthy periodontal tissues. Periodontal probing revealed a 12‐mm furcation (mid‐buccal) pocket consistent with the cyst location. (C–E) CBCT view shows an expansive lesion with a periosteal reaction on the buccal surface, a well‐defined hypodense lesion surrounding the buccal aspect of Tooth #18. The arrow symbol shows an expansive lesion with periosteal reaction on the buccal surface.(F) CBCT sagittal view shows a hypodense lesion involving the mesial root of Tooth #18.

The patient and guardian reported no significant medical history, allergies, smoking, or alcohol abuse. Written informed consent was obtained from the patient′s guardian. Clinical examination demonstrated a firm, bony expansion of the left mandibular body. No evidence of clinical decay was noted in the mandibular left quadrant (Figure 1A). The tooth in question responded to all pulp sensibility testing, including cold and electric pulp testing (EPT) (SybronEndo, California, United States), within the normal limits. Using a four‐point periodontal probing technique, with a stainless‐steel periodontal Probe #6 (CP12/Thin Williams DE Probe, Hu‐Friedy, Chicago, United States), the affected tooth exhibited purulent exudate and a 12‐mm probing depth on the mid‐buccal surface (Figure 1B). Considering the suspicion of a cracked tooth, adjunctive diagnostic tools such as Tooth Slooth fracture detector (California, United States), transillumination (SmartLite Pro, Dentsply Sirona, United States), radiographic interpretation (XDR Radiology, Cyber Medical Imaging, United States), and high‐powered magnification with a surgical operating microscope (ZEISS EXTARO 300, Carl Zeiss, Oberkochen, Germany) were utilized in an attempt to rule out a vertical root fracture.

The preoperative limited‐view cone beam computed tomography (CBCT) (Carestream Dental CS 8100 3D, Marne‐la‐Vallee, France) rendering taken of the mandibular left quadrant demonstrated a well‐rounded hypodense area in the buccal area of Tooth #18 of an expansile nature and well‐corticated (Figure 1C–E), with no evidence of root resorption and tooth displacement. The lesion within the CBCT did not appear within the epicenter of Tooth #18 after inspection of the sagittal view (Figure 1F).

The associated radiographic interpretation of the CBCT was taken in conjunction with all endodontic testing to conclude that the pulp was vital. Immediate referral to the NSU‐OMFS department enabled a precise, conservative excisional biopsy and enucleation of the lesion associated with #18.

### 2.1. Guided Surgery

At the time of consultation, it was decided to create a custom 3D surgical guide to provide a more precise surgical window for biopsy, thereby limiting potential damage to adjacent tooth structure. Intraoral scans of the patient′s maxilla and mandible were obtained and sent in‐house, along with the patient′s CBCT, to the NSU 3D print labs for fabrication of a surgical guide and 3D surgical model. The CBCT data were segmented to identify the lesion boundaries and adjacent anatomical structures, particularly the roots of neighboring teeth. Based on this segmentation, a tooth‐supported surgical guide was designed to accurately transfer the planned osteotomy location to the surgical field. The dimensions of the osteotomy window were determined based on the three‐dimensional extent of the lesion visualized on CBCT, maintaining a safety margin from adjacent roots to facilitate lesion access and enucleation while minimizing unnecessary bone removal. Prior to surgery, the surgical guide was sterilized in an autoclave.

**Figure 2:**
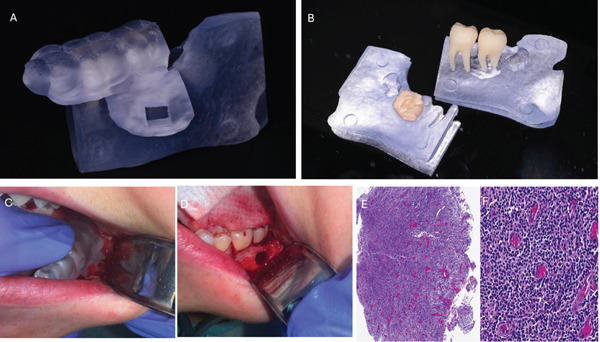
3D customized surgical guide and histologic section. (A, B) The 3D model with the surgical guide. (C) The surgical guide inserted into the mouth to confirm proper seating. (D) An osteotomy was created within the bony window superficially. The guide was then removed. (E, F) Histologic features of the mandibular buccal bifurcation cyst. The biopsy specimen shows a section through fibrovascular tissue with a very dense infiltrate of lymphocytes and plasma cells, with occasional macrophages and multinucleated giant cells. Hematoxylin–eosin staining (E) ×4 magnification and (F) ×20 magnification.

Two weeks later, the patient and her mother returned for surgery. A 3D model and surgical guide were reviewed with them to explain the procedure before it began (Figure 2A,B). The surgical guide was inserted into the mouth to confirm proper seating (Figure 2C). At this time, informed consent was obtained, and a timeout was performed. An inferior alveolar nerve (IAN) was anesthetized with one cartridge volume (1.8 mL) of 2% lidocaine with 1:100,000 epinephrine (Henry Schein, Melville, New York, United States) and one cartridge volume (1.8 mL) of 0.5% Marcaine with 1:200,000 epinephrine (Cook‐Waite, Cambridge, Canada) to achieve profound anesthesia. A #15 blade (Henry Schein, Melville, New York, United States) was used to create a distal hockey stick incision, which was then tracked mesially along the sulci of Teeth #18 and #19. A periosteal elevator (Hu‐Friedy, Chicago, United States) was used to create a full‐thickness mucoperiosteal flap. A throat pack was placed in the mouth, and the surgical guide was fastened to Teeth #18–#20. An osteotomy was created within the bony window superficially. The guide was then removed (Figure 2D), and the site was deepened lingually until the bony lesion was identified. A spoon excavator (#31W Excavator‐6 Handle, Hu‐Friedy, Chicago, United States) was used to detach the bony lesion superiorly and to remove the remaining tissue for adequate retrieval of the biopsy specimen. The site was then irrigated thoroughly with copious saline.

The surgical site was re‐evaluated. No IAN exposure or damage to adjacent structures was visualized. The surgical site was closed using 3‐0 chromic gut absorbable suture (Henry Schein, Melville, New York, United States) to approximate and secure the gingival tissue to its normal anatomical position. Adequate hemostasis was achieved. Postoperative instructions were given to both the patient and mother, who were instructed to follow up with the OMFS clinic in 1 week. The tissue specimen was placed in a plastic formalin container and sent to oral pathology for histologic evaluation.

### 2.2. Histology

Following surgical intervention, histology results revealed chronic inflammatory tissue. The biopsy specimen for the case report was nonspecific, demonstrating a section through fibrovascular tissue with a very dense infiltrate of lymphocytes and plasma cells, with occasional macrophages and multinucleated giant cells (Figure 2E,F). The inflammatory infiltrates observed in our biopsy sample of fibrovascular tissue align with those seen in an MBBC. Based on the clinical and radiographic presentation of the cyst, we can deduce that the inflammatory tissue is more specifically an MBBC.

### 2.3. Follow‐Up

**Figure 3:**
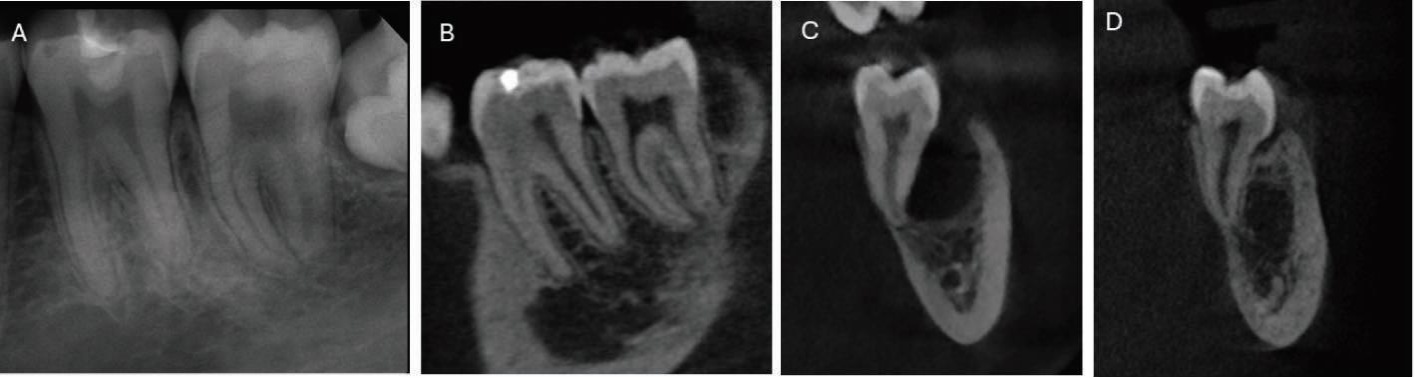
Preoperative CBCT and periapical radiograph and CBCT images at 1‐year follow‐up. (A) A postoperative periapical radiograph and (B) CBCT revealed an intact lamina dura with no signs of periapical radiolucency or pathology related to Tooth #18. (C) Preoperative CBCT coronal view shows an expansile nature of the lesion on the buccal surface. (D) Postoperative CBCT coronal view shows complete resolution of the cystic process and re‐establishment of buccal cortical and cancellous bone.

At the 1‐year follow‐up, the clinical and radiographic evaluation of Tooth #18 showed no signs or symptoms, no evidence of intraoral or extraoral swelling, no sinus tract formation, and a resolution of the prior bony expansion. Periodontal probing depths are unremarkable and within normal limits. A postoperative periapical radiograph (Figure 3A) and CBCT (Figure 3B) revealed an intact lamina dura with no signs of periapical radiolucency or pathology related to Tooth #18. The preoperative CBCT (Figure 3C) and postoperative coronal views (Figure 3D) revealed complete resolution of the cystic process and re‐establishment of buccal cortical and cancellous bone. Sensibility testing suggests that Tooth #18 has remained vital, responding positively to cold testing and exhibiting a positive EPT response, with no sensitivity to percussion or palpation. These findings are consistent with a stable postsurgical outcome following the previous diagnosis of an MBBC, with histological evidence of chronic inflammatory granulation tissue. At this time, the tooth remains vital, with normal pulp and normal apical tissues. Table 1 shows the timeline for the case report presentation.

**Table 1:** Timeline for the case report presentation.

Initial presentation	Patient referred to Oral and Maxillofacial Surgery (OMFS) evaluation for possible extraction of Tooth #18 due to a concerning radiographic lesion
Visit 1	Following surgical evaluation, the patient was referred to the Endodontic Department for further assessment. After the endodontic evaluation, an immediate referral to the OMFS department was made
Visit 2 (2 weeks later)	Fabrication of a surgical guide and 3D surgical model was performed. A conservative excisional biopsy and enucleation of the lesion associated with Tooth #18 were conducted. The specimen was sent to oral pathology for histologic evaluation
Visit 3 (1 week later)	Patient returned for re‐evaluation; sutures were removed. Histology results indicated inflammatory tissue, specifically an MBBC
Visit 4: Follow‐up (1 year later)	The clinical and radiographic evaluation of Tooth #18 revealed no signs or symptoms. Sensibility testing indicates that Tooth #18 remains vital, and the prior bony expansion has resolved

## 3. Discussion

MBBC was first described by Stoneman and Worth in 1983 as the “mandibular infected buccal cyst” [7]. This lesion has received other names, including juvenile paradental cyst (PC) and circumferential dentigerous cyst [13]. However, the World Health Organization (WHO) recommends reserving the term PC for cysts associated with mandibular third molars, whereas MBBC should specifically denote those occurring in relation to mandibular first or second molars. Both MBBC and PC are classified as collateral inflammatory cysts [14]. MBBC is an uncommon inflammatory odontogenic cyst, accounting for approximately 5% of all odontogenic cysts [15, 16]. This condition predominantly occurs in children and adolescents during the mixed or early permanent dentition stage, typically involving the buccal aspect of mandibular first or second permanent molars, with reported cases ranging from 4 to 14 years of age [17]. It is considered a variant of inflammatory odontogenic cysts and is characterized by unique clinical, radiographic, and histopathologic features that distinguish it from other mandibular cystic lesions.

Although its etiopathogenesis remains unclear, it is hypothesized that food impaction and debris accumulation within buccal periodontal pockets of tilted teeth trigger chronic inflammation of the pericoronal tissues, leading to proliferation of epithelial rests and subsequent cyst formation. This mechanism helps explain why MBBC is more commonly associated with lower molars and is uncommon in lower premolars [18]. To date, no reports have described this type of cyst affecting maxillary molars.

Clinically, patients may present with buccal swelling, delayed eruption, pain, or recurrent episodes of pericoronitis, although asymptomatic cases identified incidentally on radiographic examination have also been reported. A hallmark feature consistent with this case report is the buccal tipping of the involved tooth roots and buccal cortical expansion with preservation of the periodontal ligament space and lamina dura, which helps differentiate MBBC from lateral radicular cysts, dentigerous cysts, and odontogenic keratocyst [13, 17]. Deep periodontal pockets are present with or without purulent exudation. Diagnostic testing always reveals a vital pulp, resulting in a pulpal diagnosis of normal tissue with normal periapical tissues. Although uncommon in a 16‐year‐old patient, we can infer that the lesion grew slowly over the past 4–9 years, which aids in its correct diagnosis.

Differential diagnoses of MBBC include radicular cysts and lateral periodontal cysts. Radicular cysts have similar radiographic components but can be ruled out via sensibility testing [19]. PCs typically appear on the buccal and distal surfaces of erupted mandibular third molars and have a direct association with pericoronitis [20].

The histologic characteristics of the MBBC are nonspecific and are characterized by nonkeratinized stratified squamous epithelium with areas of epithelial hyperplasia and inflammatory infiltration [21]. Occasionally, biopsy specimens will also include neutrophils and eosinophils within the fibrous cystic capsule [19]. The inflammatory infiltrates in our biopsy specimen were nonspecific, including a section through fibrovascular tissue with a very dense infiltrate of lymphocytes and plasma cells, with occasional macrophages and multinucleated giant cells, all findings consistent with those of an MBBC. It is important to understand that most diagnoses of MBBC are established after clinical, radiographic, and histopathologic examination [19].

Management of MBBC remains a subject of discussion in the literature, with treatment options ranging from conservative monitoring to surgical intervention [6, 7–11]. Over the last decade, digital technology has been of paramount importance in improving diagnostic processes, treatment planning, and surgical procedures in dentistry, enabling the management of complex cases with predictable outcomes compared with freehand methods [22].

In the present case, one of the most innovative aspects of this approach was the design and fabrication of a 3D‐printed surgical guide to facilitate the enucleation of the MBBC. The guided surgical approach was selected to facilitate accurate transfer of the virtual surgical plan to the clinical setting, preserve tooth vitality, avoid compromising any roots during surgery, reduce intraoperative time, and prevent postoperative complications. In the present case, the lesion was located in close proximity to adjacent tooth roots, making precise osteotomy positioning essential to minimize the risk of iatrogenic damage. Compared with a conventional freehand approach, the guide enabled accurate localization of the lesion, preservation of neighboring root structures, and limitation of flap extension and bone removal to the area strictly required for access. Furthermore, guided surgery may improve surgical predictability and reproducibility, particularly in anatomically challenging cases. An additional benefit is its value as an educational and patient communication tool, as the digital planning process allows both clinicians and patients to better visualize the surgical procedure and anticipated outcomes.

Moreover, advanced imaging, particularly small‐field‐of‐view CBCT, played a crucial role in preoperative diagnosis and planning, confirming the lesion′s buccal location, assessing the extent of cortical plate expansion, and excluding lingual involvement or aggressive behavior [23].

Although the use of a surgical guide provided significant advantages in the present case, it may not be required in all cases of MBBC. Factors such as access to digital technologies, laboratory or printing facilities, and the additional planning steps required should be considered when selecting the surgical approach. Nevertheless, CBCT imaging often allows three‐dimensional evaluation of the lesion and its relationship to adjacent structures. In cases where preservation of adjacent roots and precise osteotomy localization are critical, guided surgery may offer increased accuracy and surgical predictability while reducing the extent of bone removal.

In addition, this case demonstrates the crucial role of interdisciplinary and multidisciplinary care in assisting the dental professional community in making informed decisions when patients present with contradictory or nonspecific clinical and radiographic findings. Appropriate management ultimately benefits patients by retaining teeth and maintaining vitality. The patient and guardian were satisfied with the treatment outcome, as the tooth was successfully preserved, extraction was avoided, and root canal treatment was not necessary; moreover, the patient′s preference to retain the tooth was respected and achieved.

Prognosis following appropriate management of MBBC is favorable, with recurrence being rare. Preservation of the affected molar is generally successful, and continued root development and eruption have been documented in pediatric patients following cyst removal. Long‐term follow‐up is recommended to ensure adequate bone regeneration and tooth vitality [10, 17].

## 4. Conclusion

This case report highlights the importance of considering MBBC in the differential diagnosis of radiolucent lesions associated with erupting mandibular molars in pediatric and adolescent patients. Early detection through key clinical and radiographic features, along with evidence‐based management and a guided surgical approach, is crucial for preventing misdiagnosis and unnecessary endodontic treatment. Increasing awareness of this condition among clinicians and endodontists can encourage conservative, tooth‐preserving treatment and enhance patient outcomes.

## Author Contributions

**Adrian Reyes:** investigation, data curation, resources, writing – original draft. **Ishaan Goel:** investigation, data curation, resources, writing – original draft. **Steven Kaltman:** investigation, data curation, resources. **Yehuda Benjamin:** investigation, data curation, resources, writing – review and editing. **Catalina Mendez de la Espriella:** investigation, data curation, resources, writing – review and editing. **Rodrigo R. Amaral:** conceptualization, investigation, data curation, resources, methodology, writing – original draft, supervision, project administration.

## Funding

No funding was received for this manuscript.

## Conflicts of Interest

The authors declare no conflicts of interest.

## Data Availability

Data sharing is not applicable to this article as no datasets were generated or analyzed during the current study.
